# Deciphering the Role of Ubiquitination in Tauopathies

**DOI:** 10.1002/alz.095263

**Published:** 2025-01-09

**Authors:** Axelle A.T. Vanparys, Nathalie Kyalu Ngoie Zola, Clémence Balty, Emilien Boyer, Didier Vertommen, Pascal Kienlen‐Campard, Bernard J Hanseeuw

**Affiliations:** ^1^ Institute of Neuroscience ‐ UCLouvain, Brussels Belgium; ^2^ De Duve Institute ‐ UCLouvain, Brussels Belgium; ^3^ Department of Neurology, Saint‐Luc University Hospital, Brussels Belgium; ^4^ Massachusetts General Hospital, Harvard Medical School, Boston, MA USA

## Abstract

**Background:**

Tauopathies are defined as a group of heterogeneous neurodegenerative disorders, characterized by abnormal Tau protein accumulation in neurons and glial cells. The most common tauopathy is Alzheimer’s disease (AD), which is responsible for 70% of cases dementia. The development of tauopathies is a multistep process probably driven by changes in the post‐translational modifications (PTMs) of Tau. Unfortunately, for almost all these diseases, no accurate differential diagnostic methods are available which exacerbates the need of identifying biomarkers or treatments for tauopathies. In addition to providing a better understanding of the pathophysiology of tauopathies, the study of PTMs seems well suitable for these aims. According to the literature and previous results from the lab, ubiquitination appears promising to identify biomarkers allowing the differentiation of tauopathies and to treat these pathologies. Therefore, this project aims to evaluate the predictive value of ubiquitination dysregulation in the tauopathies, as a tool for diagnosis and a mechanism of tau aggregation.

**Method:**

To achieve our goal, we first segregated the soluble and insoluble brain protein fractions from brain of subjects with specific tauopathies (AD, Pick’s disease, frontotemporal lobar degeneration and corticobasal degeneration) and healthy subjects. Tandem mass spectrometry (LC‐MS/MS) was used to identify all the proteins present in the insoluble fractions using DDA (data dependent acquisition), as well as in the soluble fraction enriched in ubiquitinated proteins. The post‐analysis was performed using Proteome Discoverer software and the statistical analyses were done in R.

**Result:**

This analysis allows the identification of an imbalance in proteins ubiquitination in the insoluble fraction of tauopathies brains. Moreover, the imbalance provides ubiquitin hallmarks associated to specific tauopathies. Finally, we also investigated Tau ubiquitination profile in these fractions.

**Conclusion:**

Our study suggests that ubiquitination may be dysregulated in tauopathies, in which ubiquitinated tau accumulates. This could arise either from impairment of tau clearance in autophagy/lysosomal compartments, or as a consequence of altered proteasome function, well‐described in AD. The dysregulation of ubiquitination appearing in tauopathies underscores the potentially significant role of this post‐translational modification in the development of these pathologies.

